# Building Personalized Cancer Therapeutics through Multi-Omics Assays and Bacteriophage-Eukaryotic Cell Interactions

**DOI:** 10.3390/ijms22189712

**Published:** 2021-09-08

**Authors:** Qing Wang

**Affiliations:** Complete Omics Inc., 1448 S. Rolling Rd, Baltimore, MD 21227, USA; qing@completeomics.com

**Keywords:** phage display, neoantigen, immunotherapeutic, cancer vaccine, personalized cancer treatment

## Abstract

Bacteriophage-eukaryotic cell interaction provides the biological foundation of Phage Display technology, which has been widely adopted in studies involving protein-protein and protein-peptide interactions, and it provides a direct link between the proteins and the DNA encoding them. Phage display has also facilitated the development of new therapeutic agents targeting personalized cancer mutations. Proteins encoded by mutant genes in cancers can be processed and presented on the tumor cell surface by human leukocyte antigen (HLA) molecules, and such mutant peptides are called Neoantigens. Neoantigens are naturally existing tumor markers presented on the cell surface. In clinical settings, the T-cell recognition of neoantigens is the foundation of cancer immunotherapeutics. This year, we utilized phage display to successfully develop the 1st antibody-based neoantigen targeting approach for next-generation personalized cancer therapeutics. In this article, we discussed the strategies for identifying neoantigens, followed by using phage display to create personalized cancer therapeutics—a complete pipeline for personalized cancer treatment.

## 1. Introduction

The human body hosts a large amount of microbes, including archaea, bacteria, fungi, viruses, and protozoa [1,2]. Among these, phages infect bacterial hosts and can trigger the lytic replication, release of new phage particles, and new bacterial infections [3]. In addition, phages can also “collaborate” with some bacterial to kill others. The famous “kill-the-winner” model demonstrated that higher-abundance bacterial species have a greater chance of encountering virulent phages and therefore suffer more dramatically than the low abundance bacterial species, which can cause a reset to balance in abundances between different bacterial species [4]. The interaction between phage and bacterial species depends on the binding between phage surface proteins and bacteria. To utilize such features for biotechnological or therapeutic purposes, Phage Display was introduced by Smith et al. in 1985 [5]. Phage display is a process in which libraries of proteins or peptides can be displayed as fusion proteins with one of the coat proteins on the phage surface [6]. Because phage display created a simple bridge between a DNA packaged with the phage and the binding targets of the phage, it provides a powerful method for identifying the strong binders over multiple rounds of selection. Phage display can be adopted in immune library screening, where a DNA library can be first introduced into phage vectors through cloning, and the subsequent screening procedure can help identify the phages that can express antibodies or a part of an antibody that can bind with a target protein molecule [7]. Most importantly, the DNA molecule encoding this antibody or antibody fragment within the phage can be characterized for further applications. Among the many applications of phage display with immune libraries, identifying antibodies that can specifically interact with cancer cells holds the most significant clinical potential.

Cancer is one of the leading causes of human death, and it is initiated from genetic mutations that alter a normal cell’s behaviors [8,9]. Proteins encoded by mutant genes can be processed into mutation-carrying peptides and presented onto cell surface through human leukocyte antigen (HLA), and such peptides are called neoantigens [10]. Neoantigens are cancer-specific biomarkers, and they not only can distinguish cancer cells from normal but also do not induce autoimmune toxicity due to their nature of bypassing central tolerance [11]. These features make neoantigens the foundation for numerous cancer immunotherapeutic approaches, including immune checkpoint inhibitors, such as PD-1, and cancer vaccines under development [11,12]. The effectiveness of immunotherapies against cancers is often remarkable, which leads to dramatic attention to neoantigen in recent years [13,14]. With the recent development of cancer genomics readily identifying patient-specific mutations, neoantigen-based personalized therapeutics is becoming feasible [15,16]. Through phage display, we successfully developed two neoantigen-targeting personalized cancer drugs and observed phenomenal therapeutic effects [17,18].

**Figure 1:**
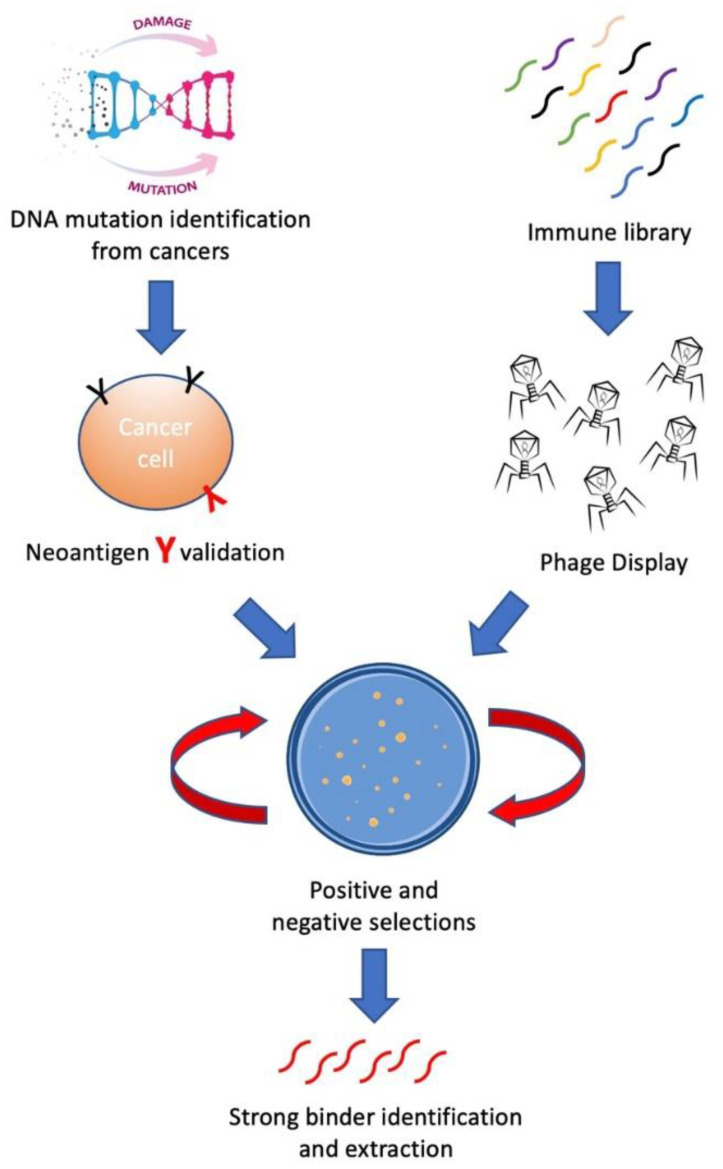
Workflow for developing personalized cancer therapeutics through accurate neoantigen validation and phage display.

In this review, we aim to introduce methods for building personalized cancer therapeutics through phage-eukaryotic cell interaction based on the correct identification of neoantigen as personalized therapeutic targets. We summarized a feasible technology pipeline bridging cancer genomics, immunotherapeutics as well as vaccine development through phage display to enable personalized cancer therapeutics (Figure 1).

## 2. Neoantigen—Personalized Cancer Therapeutic Target

With the development of numerous sequencing approaches in the past two decades, the genomic information of any biological sample is readily available through highly standardized pipelines [19]. One of the most important successes in the healthcare industry in the past decade is the commercialization of next-generation sequencing (NGS) technologies into the clinical space [20]. Advances in NGS have allowed the comprehensive analysis of a cancer patient’s genome to be completed within a couple of days under 1 thousand USD nowadays as compared to taking several years with millions of dollars in the early 2000s [21]. Such fundamental changes have made cancer genome analysis now standard care for cancer patients. Patient-specific mutations can be readily identified, thus laying a solid foundation for further individualized cancer therapeutics and management.

In an earlier generation of individualized cancer care or so-called “targeted therapeutics”, drug-targeting mutations are evaluated for the appropriate patients (a procedure called “Companion Diagnostics”) to decide the feasibility of adopting a particular drug. For example, Vemurafenib (Zelboraf^®^) targeting BRAF V600E mutant protein is prescribed to treat melanoma patients with BRAF V600E mutation, and imatinib mesylate (Gleevec^®^) only targets the patients with BCR-ABL fusion proteins in their leukemia cells [22,23]. So far, there are over 100 targeted therapeutic drugs available for a very limited number of druggable mutations [24]. Despite the current success in targeted therapeutics, there are still a large number of patients for whom their patient-specific mutations have no available targeted therapeutics. In addition, most patients who initially benefit from targeted therapies will eventually develop resistance through additional mutations and render their initial drugs ineffective [25,26,27].

Neoantigens are produced by cancer-related mutations (including cancer driver gene mutations and passenger gene mutations) and are accessible on the cell surface; therefore, they are originated from the driving force of cancer and constitute the most robust source of therapeutic cancer targets [10,12,15,26,28,29,30]. Such universality and patient specificity distinguish neoantigen-based therapeutics from other targeted therapies. Unlike traditional targeted therapies, where the major difficulty is to screen for a chemical compound or an antibody that can interact with a druggable mutation target, in developing a neoantigen-based therapy, the most critical task is to identify the neoantigen sequence presented on the cell surface. With this information, further therapeutic approaches can be developed, including building and screening for small chemical compound drugs and antibody-based drugs or, more importantly, developing cancer vaccines [15,16,29].

### 2.1. Accurate Neoantigen Identification Lays the Foundations for Personalized Cancer Treatment

#### 2.1.1. Prediction-Based Neoantigen Identification

Neoantigens represent the most personalized cancer therapeutic targets. The most important task for building up the neoantigen-based personalized cancer therapeutic method is to know the sequence and abundance of the most feasible neoantigen targets of a patient, and this critical information provides therapeutic targets for all therapeutics, including peptide vaccine, mRNA vaccine, and engineered cell therapies, etc. There are mainly three different methods to identify a neoantigen. The first method is through computation-aided prediction algorithms. With the NGS sequencing results from a tumor sample and data regarding a patient’s HLA types, possible neoantigen sequences can be predicted through numerous algorithms [31,32,33,34]. To date, there are 15 algorithms and bioinformatic platforms reported for neoantigen prediction, with the most frequently used one being NetMHC [33,35,36,37,38,39,40,41,42,43,44,45,46,47,48,49]. Numerous other algorithms are under development, and most of them with assistance from artificial intelligence (AI) [50]. It would be a very convenient way to predict the neoantigen outright with the readily available cancer genome data; however, there are major problems in algorithm-based neoantigen predictions. First, the prediction is far from accurate. This inaccuracy comes from two directions, (1) there are only less than 5% of the predicted-to-be-presented peptides are actually presented, and (2) even for the less than 5% accurately predicted neoantigens, the affinities predicted by the algorithms are not correlated to the immunogenicity of the neoantigens presented on the cell surface [31,51,52]. Secondly, it has also been shown that only a small fraction (1–2%) of mutations are able to give rise to immunogenic neoantigens [53]. Based on these limited chances of success, it is challenging to evaluate the efficacy of a neoantigen-based therapy when only a small portion of the total dosage contains effective materials, and the treatment efficacy is expected to be at least significantly compromised [54,55].

#### 2.1.2. Functional Analysis-Based Neoantigen Identification

The second method is to screen for immunogenic neoantigens that can evoke specific T-cell responses through functional analysis [56,57,58]. In this approach, tumor cells or antigen-presenting cells that are peptide-pulsed or transfected with mutation-encoding vectors are co-cultured with autologous T-cells to therefore allow the expansion of reactive T-cell clones, followed by validation procedures using tetramer staining or peptide-pulsing assays [59,60]. A major benefit in this approach is that both identifications of neoantigens and isolation of reactive T-cells that are of potential therapeutic value can be accomplished together. However, this method requires the presence of endogenous T-cell clones that can recognize the neoantigens, and such clones are either not existing or existing at an extremely low abundance level among all T-cell clonotypes. A more obvious difficulty for this approach is that it requires co-culturing of tissue cells over a relatively long period of time (~several weeks or longer); the difficulties and hefty cost coming along with the procedure made it clinically unfavorable [61].

#### 2.1.3. Directly Detecting and Quantifying Neoantigens

The third method is that neoantigen peptides can be detected and quantified through mass spectrometry, which is by far the most direct way to observe neoantigens [52]. Advances in mass spectrometry have allowed for the rapid and comprehensive analysis of a peptidome sample [62,63]. However, neoantigen identification is still one of the most challenging tasks for mass spectrometry-based peptide detection [32,52]. Collaborations among well-established mass spectrometry-based proteomics labs are formed to improve method development and data sharing for neoantigen identification [64].

There are mainly two contradicting but highly correlated approaches in mass spectrometry-based neoantigen identifications, namely, an unselective profiling-based approach and a targeted detection approach. Profiling-based proteomics assays have been dominating the major topics in proteomics fields, where over 85% of the reported projects fall into this category [65]. With the development of the Orbitrap mass spectrometers by ThermoFisher and the most recent Trapped Ion Mobility Spectrometry-Time-of-Flight (timsTOF) mass spectrometer by Bruker, the depth of mass spectrometry-based proteomics analysis is growing dramatically [66,67]. Technical advancements have allowed for an increasingly deeper profiling of the tumor immunopeptidome, and by comparing a tumor’s genomic mutation profiling and its mass spectrometry-based peptidome data, researchers can reveal possible immunogenic neoantigens [32,68,69]. Interestingly, mass spectrometry-based immunopeptidome profiling results have also been used to better optimize the neoantigen prediction models [47,48]. Despite the tremendous efforts in this field, identifying neoantigens through the unselective profiling-based mass spectrometry approach has major difficulties to solve before successful clinical applications. First, the complete dataset obtained from an immunopeptidome profiling approach is always dominated by normal or neoantigen-unrelated antigens, and the number of valid neoantigen sequences identified is minute [32]. Secondly, the current mass spectrometry-based profiling method is still an abundance-driven approach to a certain extent [70]. That being said, the detection of low-abundance targets is usually compromised by that of the high-abundance ones [71,72]. Biologically, a valid neoantigen is presented on cell surface only at low abundances, thus not sharing a fair chance to be detected along with the majority of the peptides that are presented at much higher abundances [32,52,73]. Due to these issues, the data quality of neoantigen profiling mass spectrometry assays are usually limited and suffers from a high false discovery rate (FDR) [32]. The low-quality data combined with the difficulties in functional validation render the profiling-based neoantigen identification a daunting task to fulfill. Whereas as new advancements develop in mass spectrometry, it is reasonable to expect that the depth of the analysis will gradually be improved to eventually pick up as many peptides in the sample as physics allows [74,75]. In addition, novel peptide sequencing techniques are also under development to potentially offer new opportunities in immunopeptidome profiling [76].

An emerging approach for mass spectrometry-based neoantigen identification is to conduct targeted detection [52]. Targeted mass spectrometry primarily utilizes a different type of instrument, Triple Quadrupole mass spectrometer, as opposed to the most frequently used Orbitrap or TOF mass spectrometers in proteome or peptidome profiling assays [77,78]. The unique feature of the triple quadrupole mass spectrometer is that it filters out non-targeted molecules, thus dramatically boosts up the detection sensitivity in complicated biological samples [79,80,81]. The technical term to describe a Triple Quadrupole method is Selected Reaction Monitoring (SRM) or Multiple Reaction Monitoring (MRM) [77,82,83,84,85]. Recently, Orbitrap platforms have also been re-configured to conduct SRM/MRM alike assays, as well as another detection strategy termed Parallel Reaction Monitoring (PRM) [86,87,88]. SRM/MRM was developed decades ago mainly to detect small compounds in chemistry or clinical samples [89,90]. Based on hardware advancements in Triple Quadrupole mass spectrometers, larger molecules, including peptides, can now be readily detected and quantified [91,92]. The targeted proteomics approach enables highly selective detection and accurate quantification of target peptides, and it holds promising potential for mass spectrometry-based clinical proteomics [52,93,94]. Despite the ultra-high sensitivity and specificity in SRM methods, there are three major technical difficulties in SRM-based targeted proteomics. (1) SRM method is hard to build. Unlike profiling proteomics which is usually built upon a common collision condition for all scans, an SRM method is usually composed of a set of hundreds of pre-defined parameters (transitions) where each one of them needs to be extensively optimized, and more importantly, validated in a biologically complex sample [94]. (2) SRM method may not be tolerant to changes or derivatizations on the targets. Some amino acids may undergo chemical derivatizations biologically or during sample preparation, such as oxidization or deamination, which will result in changes in the mass. If the derivatized versions of the peptide are not considered when the method is built, they will be overlooked in the detection [52]. (3) SRM method is not high throughput. So far, only a limited number of transitions (typically several hundred) can be compiled into a single scan, and it limits the number of peptides that could be reliably detected in each analytical run to less than one thousand [95]. In recent years, numerous improvements have been made to conquer these difficulties, including new optimization strategies, extensive fractionation approaches, dynamic assembly of the methods [52,93,94]. These improvements have made SRM more feasible in clinical proteomics applications.

Despite the limitations of targeted proteomics approaches, SRM and similar methods are emerging as a bridge linking readily available patient genomic information to neoantigen identification [52]. Genomic information of the patients could be readily obtained due to the current implementation level of clinical genomics assays [96,97]. With the patient-specific mutation profile, SRM methods could be established to further focus on patient-specific neoantigen targets [52,93]. The benefit of this approach is that it takes the genomics information as a prior and uses it to by-pass the interference from a large amount of disease-unrelated high abundance proteomic targets, and it only focuses on the targets that are patient-specific and clinically meaningful, such as mutant proteins and neoantigens [52,93].

## 3. Phage-Cell Interactions and Their Therapeutic Effects

### 3.1. Phage Biology and Its Applications

Phage represents a group of the most abundant vial entities on the planet. Based on their unique anti-bacterial feature, phage has been used for combating pathogenic bacteria in clinical treatments for over a century [98]. Due to the recent emerging issues with bacterial antibiotic resistance, phage therapy has become an important choice and has gained a lot more attention in the past decade [99]. In addition to its direct therapeutic applications, phage provides an easy linkage between the protein products and their genomes; therefore, they are widely used as a biotechnological tool to study protein-ligand interactions and to screen for therapeutic antibodies [100].

### 3.2. Phage Therapy through Phage-Bacteria Interaction

Antibiotics have been widely used to treat diseases due to bacterial infections [101]. However, in recent decades, it is increasingly common to identify antibiotic-resistant bacterial strains [102]. The long history of using antibiotics has been constantly imposing a selection pressure on the bacteria, and the ones that are resistant to the drugs are selected through evolution [103]. Bacteria can always acquire new antibiotic-resistant genes when new antibiotics are introduced, and such genes accumulate in bacteria to make them resistant to both old and new antibiotics [104]. Such superbugs, bacteria that are resistant to several types of antibiotics, can infect over 2 million people in the USA yearly and kill at least 23,000 [105]. We are quickly running out of options for treating a bacterial infection with antibiotics, and multi-drug resistant bacteria are on the rise. One of the mechanisms that bacteria frequently adopt to survive through antibiotics is by pumping the drugs out. Efflux pumps are one of such mechanisms responsible for the antimicrobial resistance in biofilm structures [106]. Efflux pumps are located on the surface of the bacteria and can effectively pump antibiotics out of the bacteria [107].

Phage therapy has been used to treat bacterial infections since the early 1900s, even before antibiotics were discovered [108]. Phage will selectively interact with bacteria by recognizing the surface proteins of the bacteria. Such selection power can allow phage to kill bacterial strains with a specific surface protein efficiently, which caused a selection pressure to the bacteria [109]. For example, phage specifically targeting efflux pumps can be selected and used to treat the bacteria that are resistant to antibiotics, and the treatment may selectively generate phage-resistant bacteria that do not have efflux pumps, and such bacteria can be further killed with antibiotics [110]. The trade-off between phage resistance and drug sensitivity would improve antimicrobial therapy and prolong the lifetime of current antibiotic therapies [111]. Such a strategy provided phage therapy a unique position in fighting against superbugs.

### 3.3. Phage Display and Phage-Eukaryotic Cell Neoantigen Interaction

Phage has a unique biological feature that allows it to efficiently link the genes coding the phage and the proteins presented on the surface. Such a feature makes phage an excellent tool for antibody screening. Phage display, a technique to study the protein-ligand interaction, has been widely used in laboratories. It is one of the most effective molecular diversity techniques. Phage display is based on the fact that an encapsulated library of genotypes can be directly associated with the presentation of a library of molecules on the phage surface. Phage display has been used in a variety of applications, including epitope mapping—where a library of peptide expressing phage is used to interact with a specific antibody, therefore to pinpoint the specific epitope sequence the antibody is interacting with [112]; ligand identification for receptors—similar to antibody mapping, peptides interacting with receptors can be identified [113]; protein-protein interactions—where phage can present large proteins that are potentially interacting with a known binding partner, therefore to identify the unknown binding partners and to study the mechanism of interactions [114]; directed evolution of proteins—mutations conferring binding advantages between two proteins can be studied using a phage display library containing these variants [115]; drug discovery—peptides or ligands that can interact with drug targets can be presented through phage display [116]; and antibody screening—where a large library of antibody-displaying phage can be screened for the best antibodies that can interact with the target antigens [117].

Phage display has recently been used to screen for antibodies that can directly interact with neoantigens specific to cancer cells, thus producing a powerful method for establishing novel cancer therapeutic methods [7]. The screening procedure is conceptually simple. A DNA library encoding a large amount of antibody fragments can be synthesized or obtained, and cloned into phage vectors [118]. Phage carrying a specific DNA sequence can present an antibody or antibody fragment protein on its surface and therefore facilitate potential bindings between the phage and neoantigen targets [119]. When identifying the neoantigen binder phages, there are several critical steps that need to be ensured.

It is necessary to include several rounds of positive selections to boost up the abundance of the strong binders and several rounds of negative selections to remove the weak binders or non-binders, and the arrangement of positive-negative cycles can be adjusted according to the targets [120]. Such a series of screenings can substantially improve the power of strong binder selection [7].

During the selection, it is critical to include strong competition. When identifying strong binders for neoantigens, a wild-type peptide sharing virtually the same sequence as neoantigen sequences, with the exception of a missense mutation site, can be used for competition-based negative selection [7].

The best therapeutic targets are the neoantigens that are produced by high-prevalence cancer driver genes and at the same time can be presented by high-frequency HLA alleles on the host’s cancer cell surface, such as the neoantigens derived from TP53 and K-Ras mutant proteins and presented by the predominant HLA-A2 allele [17,18].

Through phage display with multiple rounds of selection, a potential therapeutic antibody targeting neoantigen can be established. Such candidates have to be evaluated through purification and affinity measurement when they will bind with cancer cells presenting the target neoantigen [7]. Once the best clones are identified, the DNA in the phage can be extracted and used to encode antibodies that can be produced in a massive manner and adopted for cancer treatment [7]. Due to the advanced development of next-generation sequencing techniques, a phage library of stronger binders can be easily achieved [121].

Single-chain fragment variable (scFv) or Fab are popular structures that phage display routinely adopt, and they can bind a large variety of target molecules, such as small peptides, proteins, protein complex, cell receptors, and surface glycans [122]. The 2018 Nobel Prize in Chemistry was awarded to George P. Smith and Sir Gregory P. Winter for their development of phage display technologies [123]. scFVs targeting neoantigens presented on tumor cell surface have been reported to specifically treat highly personalized mutations that were first validated and quantified on the tumor cell surface through mass spectrometry [17,18]. In addition, in 2021 May, FDA just approved the 100th monoclonal antibody for therapeutic usage, and the protocols for evaluating such therapeutic agents are readily available [124]. Phage display-based scFV screening for neoantigen targets may represent the next generation of personalized cancer treatment where a generic pipeline can be adopted to rapidly generate patient-specific mutation targeting antibodies immediately through multi-omics based neoantigen sequencing and quantification [125].

Although phage therapy holds great potential for highly personalized cancer therapeutics, it has three major difficulties. First, the success of a phage-based therapy depends on the correct identification of neoantigen sequences presented on the patient’s tumor cells. So far, neoantigen validation and quantification is still not a routine laboratory procedure and require further improvements. Second, it could be tough to identify the best phage clone through multiple rounds of positive and negative selections, and success is not guaranteed. Third, once a therapeutic phage is identified, toxicity and tolerance tests must be performed to ensure the safety of the therapeutic agent.

## 4. Off-the-Shelf and Personalized Cancer Drugs Developed through Phage Display

For hotspot mutations that are shared by a large number of cancer patients, off-the-shelf antibody therapeutics targeting these neoantigens are being established by numerous lab and pharmaceutical companies [126]. The rationale behind developing such cancer therapeutics is based on the advancement of large-scale cancer genome sequencing efforts in the recent decade. As shown in Table 1, the top 100 mutations in the human genome are responsible for close to 60% (58.23%) of all human cancers.

Such highly concentrated distribution of disease-causing mutations makes it commercially feasible for pharmaceutical companies to develop, for each mutation in Table 1, mutation-specific neoantigen-based cancer treatment methods. It is still debating to describe the origins of tumors through monoclonal vs. multiclonal theories. However, neoantigen selection follows the same procedure regardless of monoclonality or multiclonality. The first step for neoantigen validation is to perform sequencing to identify all cancer-causing mutations, followed by ranking the mutations by allele frequency. Only the top-ranked mutations will be chosen for neoantigen validation because they represent the early events in a tumor’s development regardless of the mono- or multi- clonality.

In addition to the hotspot mutations, there are also patient-specific mutations which account for a significant part of the disease-causing mutations. To treat the disease caused by such less frequent mutations, a rapid pipeline including neoantigen validation and phage display for antibody screening can be established. It is foreseeable in the near future that, immediately after the initial diagnosis of a cancer, a patient will have a small amount of cancer tissue harvested through biopsy, and the tissue will be analyzed for personalized mutations and neoantigens. Once the neoantigen sequence and abundance are determined within a couple of days after the diagnosis, personalized cancer therapeutic agents, such as scFV, can be established through phage display within several days through rapid selection cycles. Highly personalized cancer therapy can therefore be established for each cancer patient in a timely manner.

## 5. Conclusions

Bacteriophage-eukaryotic cell interaction can facilitate the interaction between phage and its target protein, for here, the protein complex containing neoantigen sequences. Phage display thus allows us to rapidly develop personalized cancer therapeutic antibodies after specifically identifying the neoantigen sequences through mass spectrometry-based neoantigen peptide targeted validation. Phage display facilitated neoantigen-based personalized cancer therapeutics, and cancer vaccinations are becoming available and will lead to a new revolution in ultimately personalized cancer therapeutics and cancer management. Technical advancements in the rapid selection of phage display and neoantigen sequence identification are desperately needed to enable rapid developments in this field.

## Figures and Tables

**Table 1:** Top 100 Cancer Driver Mutations.

DNA Change	Type	Consequences	Percentage in Cancer Patients
chr7:g.140753336A>T	Substitution	Missense BRAF V600E	4.93%
chr2:g.208248388C>T	Substitution	Missense IDH1 R132H	3.15%
chr12:g.25245350C>T	Substitution	Missense KRAS G12D	2.61%
chr3:g.179218303G>A	Substitution	Missense PIK3CA E545K	2.34%
chr3:g.179234297A>G	Substitution	Missense PIK3CA H1047R	2.22%
chr12:g.25245350C>A	Substitution	Missense KRAS G12V	2.06%
chr3:g.179218294G>A	Substitution	Missense PIK3CA E542K	1.50%
chr17:g.7675088C>T	Substitution	Missense TP53 R175H	1.49%
chr1:g.114713908T>C	Substitution	Missense NRAS Q61R	1.44%
chr17:g.7674220C>T	Substitution	Missense TP53 R248Q	1.16%
chr17:g.7673803G>A	Substitution	Missense TP53 R273C	1.13%
chr1:g.114713909G>T	Substitution	Missense NRAS Q61K	0.97%
chr12:g.25245351C>A	Substitution	Missense KRAS G12C	0.97%
chr12:g.25245347C>T	Substitution	Missense KRAS G13D	0.95%
chr1:g.6197725delT	Deletion	Frameshift RPL22 K15Rfs*5	0.93%
chr17:g.7673802C>T	Substitution	Missense TP53 R273H	0.91%
chr17:g.7674221G>A	Substitution	Missense TP53 R248W	0.89%
chr17:g.58357800delC	Deletion	Frameshift RNF43 G659Vfs*41	0.89%
chr17:g.7673776G>A	Substitution	Missense TP53 R282W	0.83%
chr17:g.7674894G>A	Substitution	Stop Gained TP53 R213*	0.72%
chr6:g.167003333delT	Deletion	Intron FGFR1OP	0.69%
chr12:g.25227341T>G	Substitution	Missense KRAS Q61H	0.66%
chr17:g.7674872T>C	Substitution	Missense TP53 Y220C	0.65%
chr10:g.87965537delT	Deletion	3 Prime UTR PTEN	0.64%
chr3:g.179199088G>A	Substitution	Missense PIK3CA R88Q	0.62%
chr1:g.64841314delT	Deletion	Frameshift JAK1 K860Nfs*16	0.57%
chr12:g.25245351C>G	Substitution	Missense KRAS G12R	0.54%
chr17:g.7674945G>A	Substitution	Stop Gained TP53 R196*	0.53%
chr17:g.20204950delA	Deletion	Frameshift SPECC1 N303Tfs*63	0.50%
chr2:g.208248389G>A	Substitution	Missense IDH1 R132C	0.49%
chr10:g.87933148G>A	Substitution	Missense PTEN R130Q	0.47%
chr14:g.104780214C>T	Substitution	Missense AKT1 E17K	0.47%
chr9:g.21971121G>A	Substitution	Stop Gained CDKN2A R80*	0.45%
chr12:g.25245350C>G	Substitution	Missense KRAS G12A	0.45%
chr17:g.7674230C>T	Substitution	Missense TP53 G245S	0.42%
chr10:g.87933147C>G	Substitution	Missense PTEN R130G	0.41%
chr5:g.112839942C>T	Substitution	Stop Gained APC R1450*	0.41%
chr17:g.7675076T>C	Substitution	Missense TP53 H179R	0.41%
chr5:g.159099589delT	Deletion	5 Prime UTR EBF1	0.39%
chr10:g.87957915C>T	Substitution	Stop Gained PTEN R233*	0.37%
chr3:g.179234297A>T	Substitution	Missense PIK3CA H1047L	0.34%
chr4:g.152328233G>A	Substitution	Missense FBXW7 R465C	0.33%
chr7:g.140753337C>T	Substitution	Missense BRAF V600M	0.33%
chr8:g.102277121delT	Deletion	Frameshift UBR5 E2121Kfs*28	0.33%
chr17:g.7670685G>A	Substitution	Stop Gained TP53 R342*	0.33%
chr16:g.67611435_67611436insA	Insertion	Frameshift CTCF T204Nfs*26	0.33%
chr14:g.55684263delA	Deletion	3 Prime UTR KTN1	0.32%
chr12:g.25245351C>T	Substitution	Missense KRAS G12S	0.32%
chr17:g.7673704G>A	Substitution	Stop Gained TP53 R306*	0.32%
chr4:g.1801841C>G	Substitution	Missense FGFR3 S249C	0.32%
chr3:g.179203765T>A	Substitution	Missense PIK3CA N345K	0.31%
chr17:g.7674953T>C	Substitution	Missense TP53 H193R	0.30%
chr17:g.7675143C>A	Substitution	Missense TP53 V157F	0.30%
chrX:g.77508202delA	Deletion	3 Prime UTR ATRX	0.30%
chr7:g.55191822T>G	Substitution	Missense EGFR L858R	0.30%
chr17:g.39711955C>T	Substitution	Missense ERBB2 S310F	0.30%
chr1:g.114716124C>G	Substitution	Missense NRAS G13R	0.30%
chr19:g.3118944A>T	Substitution	Missense GNA11 Q209L	0.30%
chr1:g.26779440delG	Deletion	Frameshift ARID1A D1850Tfs*33	0.29%
chr1:g.26779863C>T	Substitution	Stop Gained ARID1A R1989*	0.29%
chr5:g.112840254_112840255insA	Insertion	Frameshift APC T1556Nfs*3	0.29%
chr19:g.52212718C>G	Substitution	Missense PPP2R1A P179R	0.28%
chr2:g.222201320delT	Deletion	Intron PAX3	0.28%
chrX:g.40062191T>C	Substitution	Missense BCOR N1459S	0.28%
chr1:g.114716126C>T	Substitution	Missense NRAS G12D	0.27%
chr4:g.152328232C>T	Substitution	Missense FBXW7 R465H	0.27%
chr10:g.87933147C>T	Substitution	Stop Gained PTEN R130*	0.26%
chr17:g.7675994C>A	Substitution	Splice Region TP53 T125T	0.26%
chr3:g.179221146G>A	Substitution	Missense PIK3CA E726K	0.26%
chr17:g.7675124T>C	Substitution	Missense TP53 Y163C	0.26%
chr5:g.158698822delA	Deletion	3 Prime UTR EBF1	0.26%
chr12:g.132676598G>C	Substitution	Missense POLE P286R	0.26%
chr1:g.114713908T>A	Substitution	Missense NRAS Q61L	0.26%
chr12:g.4301917delT	Deletion	3 Prime UTR CCND2	0.25%
chr12:g.49040709delG	Deletion	Frameshift KMT2D P2354Lfs*30	0.25%
chr10:g.87958013delA	Deletion	Frameshift PTEN K267Rfs*9	0.25%
chr10:g.87961042delTACT	Deletion	Frameshift PTEN T319*	0.24%
chr14:g.65076348delA	Deletion	3 Prime UTR MAX	0.24%
chr9:g.77794572T>G	Substitution	Missense GNAQ Q209P	0.24%
chr11:g.533874T>C	Substitution	Missense HRAS Q61R	0.24%
chr3:g.179199690G>A	Substitution	Missense PIK3CA G118D	0.24%
chr13:g.39343745delT	Deletion	3 Prime UTR LHFP	0.24%
chr17:g.7673802C>A	Substitution	Missense TP53 R273L	0.24%
chr17:g.7675085C>A	Substitution	Missense TP53 C176F	0.23%
chr10:g.121520163G>C	Substitution	Missense FGFR2 S252W	0.23%
chr9:g.21971187G>A	Substitution	Stop Gained CDKN2A R58*	0.23%
chr7:g.91973771delA	Deletion	Frameshift AKAP9 K39Rfs*17	0.23%
chr2:g.60458275delT	Deletion	3 Prime UTR BCL11A	0.23%
chr4:g.152326137G>C	Substitution	Missense FBXW7 R505G	0.23%
chr12:g.25225628C>T	Substitution	Missense KRAS A146T	0.23%
chr17:g.7674947A>G	Substitution	Missense TP53 I195T	0.23%
chr5:g.112838220C>T	Substitution	Stop Gained APC R876*	0.22%
chr17:g.7674957G>A	Substitution	Stop Gained TP53 Q192*	0.22%
chr4:g.105240988delT	Deletion	Intron TET2	0.22%
chr17:g.7674216C>A	Substitution	Missense TP53 R249S	0.22%
chr3:g.181713439delA	Deletion	3 Prime UTR SOX2	0.22%
chr3:g.41224622C>T	Substitution	Missense CTNNB1 S37F	0.22%
chr17:g.7673767C>T	Substitution	Missense TP53 E285K	0.22%
chr5:g.112838934C>T	Substitution	Stop Gained APC R1114*	0.22%
chr17:g.7675085C>T	Substitution	Missense TP53 C176Y	0.22%
		Total	58.23%
